# Density Peaks Clustering by Zero-Pointed Samples of Regional Group Borders

**DOI:** 10.1155/2020/8891778

**Published:** 2020-07-18

**Authors:** Lin Ding, Weihong Xu, Yuantao Chen

**Affiliations:** ^1^School of Computer and Communication Engineering and Hunan Provincial Key Laboratory of Intelligent Processing of Big Data on Transportation, Changsha University of Science and Technology, Changsha, Hunan 410114, China; ^2^School of Computer Science and Engineering, Nanjing University of Science and Technology, Nanjing, Jiangsu 210094, China

## Abstract

Density peaks clustering algorithm (DPC) has attracted the attention of many scholars because of its multiple advantages, including efficiently determining cluster centers, a lower number of parameters, no iterations, and no border noise. However, DPC does not provide a reliable and specific selection method of threshold (cutoff distance) and an automatic selection strategy of cluster centers. In this paper, we propose density peaks clustering by zero-pointed samples (DPC-ZPSs) of regional group borders. DPC-ZPS finds the subclusters and the cluster borders by zero-pointed samples (ZPSs). And then, subclusters are merged into individuals by comparing the density of edge samples. By iteration of the merger, the suitable dc and cluster centers are ensured. Finally, we compared state-of-the-art methods with our proposal in public datasets. Experiments show that our algorithm automatically determines cutoff distance and centers accurately.

## 1. Introduction

Clustering algorithm [1], as the unsupervised learning method, divides the objectives that also are called elements, samples, and items, into several groups according to the similarity of objectives. Compared with supervised learning [2–16], it can carry out the grouping task even though the category labels are pending. Hence, it is widely used in image segmentation [17], bioinformatics [18], pattern recognition [19], data mining [20], and other fields [21, 22]. Representative clustering algorithms cover K-means [23, 24] and fuzzy c-means [25, 26] based on partitioning; AGNES [27], BIRCH [28, 29], and CURE [30, 31] based on hierarchy; DBSCAN [32] and OPTICS [33] based on density; STING [34] based on grids; and statistical clustering CMM [35] and spectral clustering [36] based on graph theory [37]. K-means is extremely sensitive to noise and the selection of the initial clustering centers, and the number of clusters needs to be set a priori. Similarly, fuzzy c-means suffers from initial partition dependence, noise, and outliers. The hierarchical clustering requires to determine the number of clusters a priori, and its effect depends on the choice of distance measurement of groups. Density-based DBSCAN, OPTICS, and grid-based clustering algorithms determine the number of clusters without artificial intervention. Still, all require preset parameters epsilon and minpts, and a mass of argument adjustments were taken to obtain optimal clustering results. These two types of algorithms generate noises around the cluster boundaries. Statistics-based CMM needs to select one or more suitable probability models to fit a dataset.

Clustering by fast search and find of density peaks [38] was published in Science, by the preset threshold (cutoff distance, dc), manually selecting the cluster centers from the decision graph proposed by DPC. Compared with traditional clustering algorithms, it has many advantages, such as higher efficiency in finding cluster centers, fewer parameters, no iteration, no noise around the cluster border, and others. However, the algorithm still has the following defects:

1. The original DPC does not provide a reliable and specific selection method of dc. Hence, the cutoff distance is computed in different ways depending on the size of datasets, in which the inappropriate dc leads to performance degradation [39]. Moreover, the dc is generally challenging to determine since the range of each attribute is unknown in most cases [40].
2. It is hard to manually select the cluster centers from a dataset with a large number of clusters. And the artificial option for cluster centers cannot meet the system with high timeliness.

To overcome the above defects, many scholars proposed improvements in the original DPC algorithm. Xie et al. proposed a local density metric based on fuzzy weighted *k*-nearest neighbors to solve the problem of difficult to determine dc in the DPC algorithm [39]. Liu et al. proposed shared-nearest-neighbor-based clustering by fast search and find of density peaks clustering (SNN-DPC), which converts cutoff distance to the number of nearest neighbors [40]. Mehmood presented a nonparametric method for DPC via heat diffusion for estimating the probability distribution of a given dataset [41]. Guo et al. used linear regression to fit the decision values with a given dc and selected the elements above the fitting function as the central elements [42]. Ding et al. proposed an algorithm based on the generalized extreme value distribution (GEV) to fit the decision values in descending order [43]. In order to reduce the time complexity, an alternative method based on density peaks detection using Chebyshev inequality (DPC-CI) was also given. Ni et al. presented the concepts of density path and density gap, as well as a new threshold called dc percentage in [44]. The density gaps are used to draw the summary graph of density gaps calculated by several dc percentages. Instead of the decision graph, the appropriate threshold value is determined by manually observing the summary graph. The algorithm is able to reduce the negative impact of inappropriate dc on the clustering result.

However, in [39–41, 44–47], it is necessary to select the centers or observe the summary graph of density gaps, with the human operation. Gu et al. [42] and Ding et al. [43] proposed the strategies of automatic center selection for the original DPC, but they depend on the given appropriate dc. However, Xie et al. [39] and Liu et al. [40] showed that it was challenging to select the proper dc.

In this paper, we propose the density peaks clustering by zero-pointed samples (DPC-ZPSs) of regional group borders. Our method not only determines the suitable range of dc and the center of each cluster but also reduces the negative impact caused by manual participation in the clustering process. The main innovations and contributions in our algorithm are as follows:

1. To merge the local clusters into individuals, we present a cluster merging strategy based on comparing density among elements of two cluster borders.
2. In order to find the border of each cluster, we propose two conceptions: neighboring cluster border (NCB) and pure cluster border (PCB).
3. For the determination of the correct number of clusters, we provide an iterative procedure, which can converge dc to a suitable value.

The remainder of this paper comprises four sections: Section 2 describes the details of the original DPC and our proposal; Section 3 presents the clustering results on our method and related works and discusses the impact and value range of the parameter of DPC-ZPS; in the final section, we have a summary of the contributions and features of this paper and put forward to future work.

## 2. Materials and Methods

### 2.1. The Original DPC Algorithm

For a given dataset *X*={*x*_1_,  *x*_2_,…,  *x*_*n*_}, where *x*_*i*_={*x*_*i*1_,  *x*_*i*2_,…,  *x*_*im*_}, *i*=1,  2,…, *n*.

DPC is based on an assumption where each cluster center has a higher local density than other elements and is far from each other. Centers are manually selected using a decision graph with the local density as the abscissa and *δ*_*i*_ as the ordinate. DPC algorithm provides two methods for calculating the local density for each element of the given dataset and is expressed in equations (1) and (2). *δ*_*i*_ is calculated by equation (3):

(1) $$\begin{array}{c} \rho_{i}=\sum_{j}ℵ\left(d_{ij}-\text{dc}\right), \end{array}$$

(2) $$\begin{array}{c} \rho_{i}=\sum_{j}\exp\left(-{\left(\frac{d_{ij}}{\text{dc}}\right)}^{2}\right), \end{array}$$

(3) $$\begin{array}{c} \delta_{i}=\left\{\begin{array}{cc} if\;\exists j\;s.t.\;\rho_{i}<\rho_{j},\\ \underset{j}{\max}\left(d_{ij}\right), & \text{otherwise,} \end{array}\right. \end{array}$$

where *d*_*ij*_ is the Euclidean distance between elements *i* and *j* and dc is the cutoff distance. As shown in equation (3), *δ*_*i*_ is the minimum distance between elements *i* and *j* whose density is higher than *i*. Moreover, for *i* with the highest density, its *δ*_*i*_ is the maximum distance between *i* and *j*.

Meanwhile, to simplify the selection of centers, DPC provides the decision value *γ*_*i*_ as follows:

(4) $$\begin{array}{c} \gamma_{i}=\rho_{i}\times \delta_{i}. \end{array}$$

After the cluster centers are determined, each of the remaining samples is assigned to the nearest denser one. And the assignment is recorded in the process of calculating *δ*_*i*_.

### 2.2. Our Method

The main process of DPC-ZPS is to select multiple distances as dc at equal intervals and calculate the corresponding decision values. Then, among the decision values of each group, the elements greater than the sum of the mean and standard deviation of the decision values are selected as the potential centers. In the range of multiple groups of dc, the iterative merging process makes the number of clusters close to the real value gradually.

#### 2.2.1. Related Concepts

**Definition 1 .**

(zero-pointed sample). in the assignment, each sample is assigned to the nearest denser one. And the zero-pointed sample (ZPS) is the one without any subordinates.

When dc is fixed, we use an array that consists of *n* zero units to store the assignment process. And the indexes of the array represent the sequence number of objectives. Let array(*i*) = *j*, in which sample *j* is the nearest and has density more significant than sample *i*. And cluster centers and potential cluster centers are not assigned. Subsequently, the array is broken at the zero units; then, |*C*| trees can be obtained, and each tree is a cluster.

**Definition 2 .**

(initial border). in a cluster tree, the initial border (IB) consists of all leaf nodes and their father nodes.

**Figure 1:**
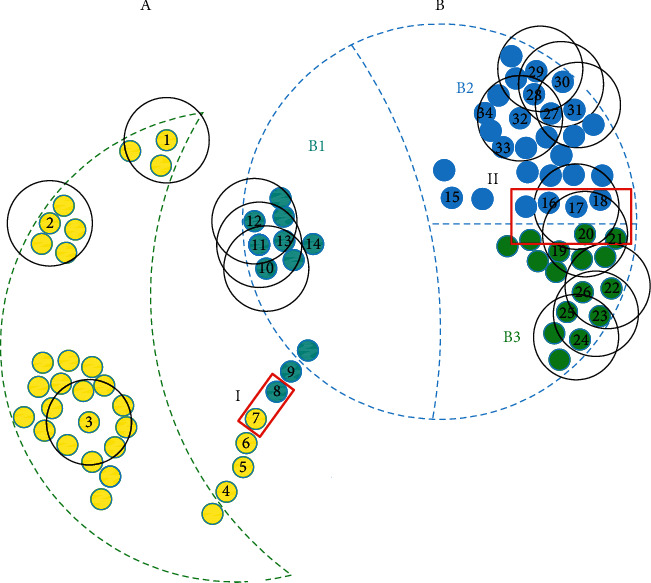
A schematic diagram of the distribution of the dataset, which only shows the distribution of a part of elements. The dashed lines represent two cluster borders, and the diameter of the solid black circle is dc.

As shown in Figure 1, elements 1, 7, and 8 are zero-pointed and leaf nodes because they are less dense than neighboring elements. Elements 3 and 32 are inner, but they are still the zero-pointed elements since they have no adjacent samples. And there are assignment paths of items 10 ⟶ 11 ⟶ 13 and 12 ⟶ 11 ⟶ 13.

**Definition 3 .**

(neighboring cluster border). clusters in a dataset *X* are denoted as *C*={*C*_*v*_|*v*=1,2,…, |*C*|}, where |*C*| is the number of clusters in *C* and *C*_*v*_={*c*_*vl*_|*l*=1,2,…, |*C*_*v*_|} ∀  *c*_*vl*_, *c*_*v*′*l*′_, where *v* ≠ *v*′, *l*′={1,2,…, |*C*_*v*′_|}, satisfies the following equation, and then *c*_*vl*_, *c*_*v*′*l*′_ ∈ NCB(*C*_*v*_,  *C*_*v*′_):

(5) $$\begin{array}{c} d\left(c_{vl},c_{v^{\prime }l^{\prime }}\right)<\bar{\bar{\text{dc}}}\left[\text{floor}\left(\text{DF}\cdot \frac{n_{vv^{\prime }}\cdot \left(n_{vv^{\prime }}-1\right)}{2}\right)\right], \end{array}$$

(6) $$\begin{array}{c} n_{vv^{\prime }}=\left|C_{v}\right|+\left|C_{v^{\prime }}\right|, \end{array}$$

where *d*( *c*_*vl*_, *c*_*v*′*l*′_) is the distance between *c*_*vl*_ and *c*_*v*′*l*′_, $\bar{\bar{\text{dc}}}$ is an array storing all *d*( *c*_*vl*_, *c*_*v*′*l*′_) of cluster pair *C*_*v*_ and *C*_*v*′_ in descending order, $\bar{\bar{\text{dc}}}\left[a\right]$ represents the *a*^th^ distance, DF is the depth factor of the neighboring cluster-border, its range is (0,1], and floor(*b*) is the integer part of *b*.

Neighboring cluster border (NCB) consists of all NCB(*C*_*v*_,  *C*_*v*′_), and it is expressed as follows, where *v* < *v*′ is to delete the symmetrical cluster pairs:

(7) $$\begin{array}{c} \text{NCB}=\underset{v<v^{\prime }}{\cup }\text{NCB}\left(C_{v},\;C_{v^{\prime }}\right). \end{array}$$

It is necessary that two clusters are far from each other with an enormous DF to attain a nonblank NCB. And the bigger the required DF value of the nonblank NCB is, the further distance the two clusters are. While for neighboring subclusters, DF is relatively minute. In the fourth chapter, the DF will be compared with parameters of DPC and is discussed to show the impact on the clustering result.

As shown in Figure 1, there are two clusters A and B in a dataset, and cluster B is misclassified into B1, B2, and B3. The elements I, 7 and 8, and II, 16, 17, 18, 19, 20, and 21, are marked with red wireframes. They belong to NCB.

**Definition 4 .**

(pure cluster border). in a cluster, the pure cluster border (PCB) is defined by the following equation:

(8) $$\begin{array}{c} \text{PCB}=\text{initial border}-\left(\text{initial border}\cap^{}\text{NCB}\right),\\ {\text{PCB}}_{v}=\text{PCB}\cap^{}C_{v}. \end{array}$$

**Figure 2:**
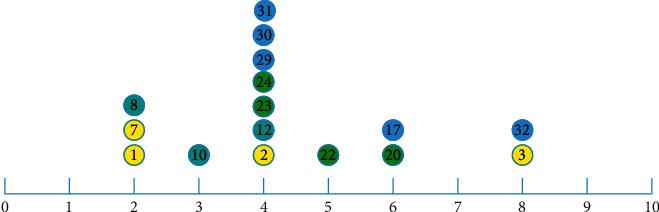
Density of parts of elements in initial borders calculated by equation (1).

Correspondingly, elements 1, 2, 4, 5, 6, 9, 10, 11, 12, 22, 23, 24, 29, 30, and 31 belong to pure cluster border (PCB) of respective clusters. However, as shown in Figure 2, elements 3 and 32 are zero-pointed since they are relatively isolated, but their density is much larger than other ZPS.

To filter out interior and isolated ZPS, we use the three-point method in fuzzy math to measure the three memberships of the elements in the PCB_*v*_, including “low density,” “medium density,” and “high density.” In order to prevent the extreme value of elements density from affecting the membership value, we select the normal distribution function as the membership function, and three functions are expressed as follows:

(9) $$\begin{array}{c} D1\left(x\right)=\exp\left(-{\left(\frac{x-\min_{f\in {\text{PCB}}_{v}}\left(\rho_{f}\right)}{\sigma }\right)}^{2}\right), \end{array}$$

(10) $$\begin{array}{c} D3\left(x\right)=\exp\left(-{\left(\frac{x-\max_{f\in {\text{PCB}}_{v}}\left(\rho_{f}\right)}{\sigma }\right)}^{2}\right), \end{array}$$

(11) $$\begin{array}{c} D2\left(x\right)=1-D1\left(x\right)-D3\left(x\right), \end{array}$$

where *σ* is the standard deviation of the density values of all elements in PCB_*v*_.

**Figure 3:**
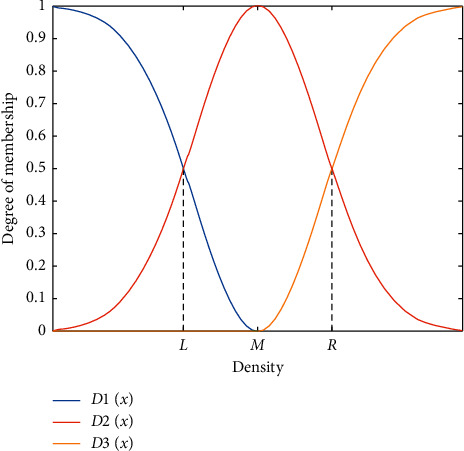
A schematic diagram of *D*1(*x*), *D*2(*x*), and *D*3(*x*).

In Figure 3, when *ρ* ∈ (0,  *M*), the membership of the element is smaller acute-angle border element than a higher density. For example, element 1 is an acute-angular border element, and elements 2, 12, and 23 belong to obtuse-angular border elements. When *ρ*  =  *L*, the degrees of two memberships are equal. When *ρ* ∈  (*M*, max_*f*∈PCB_*v*__(*ρ*_*f*_)], the higher the element density is, the smaller the membership degree of the element is, which is an obtuse-border element, and the higher the membership degree of the independent objective within the cluster. When *ρ* = *R*, the two memberships are equal.

#### 2.2.2. Merger Strategy

If a real cluster is mistakenly divided into several subclusters, there are some zero-pointed elements in the NCB since the NCB is not only the inner part of the actual group but also the border of subclusters. Due to the aggregation of zero-pointed objectives in the NCB, the density of NCB elements is smaller than other inner parts, which corresponds to *ρ* ∈ (*M*, *R*) in Figure 3. Meanwhile, the density of PCB is in *ρ* ∈ (0,  *M*). We propose a merging strategy based on the comparison of element density values of NCB and PCB.

If ∃ $c_{v_{{\hat{l}}^{\prime }}},c_{v\prime {}_{{\hat{l}}^{\prime }}}\;\in \text{NCB}\;\left(C_{v},\;C_{v\prime }\right)$satisfies $\rho_{c_{v_{\hat{l}}}}>\max_{c_{v_{l}}\in {\text{PCB}}_{v}}\rho_{c_{v_{l}}}$ and $\rho_{c_{v_{{\hat{l}}^{\prime }}^{\prime }}}>\max_{c_{v^{\prime }l^{\prime }}\in {\text{PCB}}_{v^{\prime }}}\rho_{c_{v^{\prime }l^{\prime }}}$, where max_*c*_*v*_*l*__∈PCB_*v*__*ρ*_*c*_*v*_*l*___ and max_*c*_*v*′_*l*_′_∈PCB_*v*′__*ρ*_*c*_*v*′_*l*_′__ are equal to respective *M*, then *C*_*v*_ *and* *C*_*v*′_ are merged; namely, if the density of the elements of the NCB is not more prominent than *R* but more significant than *M*, they must be the inner elements of the real cluster.

#### 2.2.3. The Iteration Strategy

**Figure 4:**
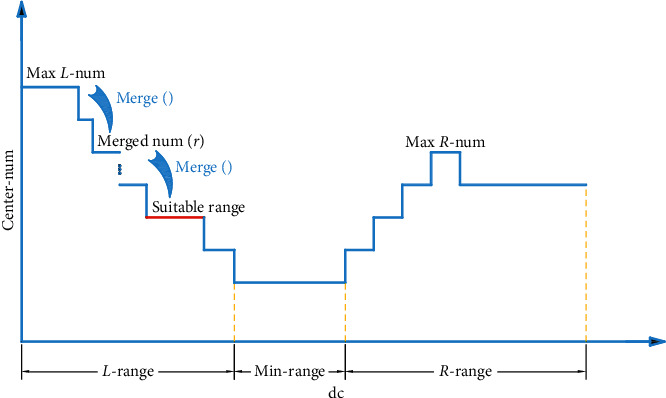
The ordinate is the number of cluster centers, and the abscissa is the dc; the min-range corresponds to the minimum number of centers. The left side of min-range is the left subrange (*L*-range), and the right side of min-range is the right subrange (*R*-range); max *L*-num and max *R*-num are the maximum numbers of cluster centers in the left and right subranges, respectively; the red line is the suitable range of dc.

The *δ* value of each center depends on the minimum distance between the central objectives and the more significant density objectives. But when the dc is small and far from its suitable range, the algorithm does not measure the density of each sample accurately and precisely. The inexact measurement shows that, in some clusters, local center elements with more prominent local density and far from the suitable center of each group are selected, and their *δ* values are much larger than noncenter items. With the increase in dc, the density measurement capability gradually strengthens. The DPC-ZPS algorithm sequentially filters out fake centers with the weakest central attributes until dc ∈ suitable range. When dc is bigger than the most significant value of the suitable range, the clusters with smaller distribution areas will be filtered out; namely, there is not the center selected by the threshold. When dc continues to increase, in the groups with a larger distribution area, the fake centers will appear again. Essentially, the process of dc increase is a gradual transition of the density metric to measure the universal density of elements from their local density. This change process is generally shown in Figure 4.

Based on the above analysis, we propose an automatic iteration strategy as follows:

  Step 1: as shown in Figure 4, after counting cluster center combination and centers quantity of each dc, the algorithm determines the min-range and divides the rest into *L*-range and *R*-range. If the min-range is not only one, the DPC-ZPS chooses the biggest one to separate the dc range.
  Step 2: let the algorithm find the max *L*-num and record its center combination as well as the sequence number of its dc.
  Step 3: according to the center combination and dc, the noncenter elements are assigned to the closest element among the denser elements.
  Step 4: execute merge() with clusters of clustering result from step 3.
  Step 5: if the number of clusters after merge() does not change, the clustering result and the number of clusters are stores; if the number of groups reduces to merged num(*r*+1) from merged num(*r*), the third to fifth steps are repeated with the center combination corresponding to the merged num(*r*+1).
  Step 6: the second to fifth steps are performed in the R-range after finding the max R-num.
  Step 7: the final result is the maximum value of the final number of clusters in two subranges and its clustering results stored by step 5.

#### 2.2.4. Time Complexity Analysis

Suppose that the number of samples in a dataset is *n*, the max center-num is *N*, the number of pairwise points in SNB is *n*_*s*_, the max center-num in dc domain is *N*^*t*^, and the number of zero-pointed samples is *n*_0_. Just like DPC, our method needs time complexity *O*(*n*^2^) to calculate the distance matrix D. We search the nearest denser neighbor for each sample via a K-D tree. And the complexity of building the K-D tree is *O*(*n* log *n*). Searching nearest neighbor queries has an average running time of *O*(log *n*), and hence, for *n* groups of dc, the complexity of searching nearest neighbor of each sample queries is *O*(*n*^2^ log *n*). For the determination of NCB, we need a matrix *M*, and the rows and columns represent the samples of two clusters. In the matrix *M*, each cell stores the distance from matrix *D*, and then, all distances in the *M* are sort in ascending order to find the NCB by equation (5). Therefore, the time complexity of NCB depends on the assignment to *M*, the times of assignment of the matrix M are 0.5(*N*^*t*^)(*N*^*t*^ − 1), the average cost is *O*(2*n*/*N*^*t*^), and the total time complexity is *O*((*N*^*t*^ − 1)*n*). How many times the operation for PCB is to be done depends on the number of zero-pointed samples, so the time complexity is less than *O*(*n*). In the merger process, the density of each pairwise points is compared, and hence, the complexity of the merger depends on the number of pairwise points in SNB and is *O*(0.5*n*_*s*_(*n*_*s*_ − 1)), where *n*_*s*_ ∈ [0,0.5*n*(*n* − 1)], and only when DF = 1, *n*_*s*_=0.5*n*(*n* − 1). However, the reasonable range of DF is (0, 0.05], which will be discussed in Section 3.3. Therefore, the time complexity of the merger is far less than *O*(0.5*n*(*n* − 1)). And iteration is based on the max center-num, and *n* ≫ *N*. We can conclude that the time complexity of the entire algorithm is *O*(*n*^2^ log *n*).

## 3. Results and Discussion

We tested our algorithm and several related works, including PPC [44], DPC [38], DBSCAN [32], OPTICS [33], and AP [54], on several datasets. These datasets have different numbers of samples and stimulate different element distributions. The detailed information is shown in Table 1. Like DPC, AP (affinity propagation) is another advanced clustering algorithm published in *Science*. The basic idea of the AP algorithm is to treat all data points as potential cluster centers (called exemplar), then connect the data points in pairs to form a network (similarity matrix), and finally transmit the information (responsibility and availability) of each edge in the network to calculate the cluster center of each sample.

### 3.1. Evaluation Criteria, Parameters of Each Algorithm, and Code Sources and Preprocessing

#### 3.1.1. Evaluation Criteria

For intuitive comparison, we chose the adjusted Rand index (ARI) [55] and adjusted mutual information (AMI) [55] to evaluate the clustering results.

The ARI formula is shown as follows:

(12) $$\begin{array}{c} \text{ARI}=\frac{\text{RI}-E\left[\text{RI}\right]}{\text{MAX}\left\{\text{RI}\right\}-E\left[\text{RI}\right]}, \end{array}$$

where *E* [RI] represents the expectations of RI. RI is calculated as follows:

(13) $$\begin{array}{c} \text{RI}=\frac{\text{TP}+\text{TN}}{C_{n}^{2}}, \end{array}$$

where TP indicates the true positive, TN indicates the real negative, and *C*_*n*_^2^ is the total number of sample pairs in a dataset containing *n* samples.

The AMI formula is shown as follows:

(14) $$\begin{array}{c} \text{AMI}=\frac{\text{MI}\left(U,V\right)-E\left[\text{MI}\left(U,V\right)\right]}{\text{MAX}\left\{H\left(U\right),H\left(V\right)\right\}-E\left[\text{MI}\left(U,V\right)\right]}, \end{array}$$

where *H*(*U*)=∑_*i*=1_^|*U*|^*P*(*i*)log_2_ *P*(*i*), *H*(*V*)=∑_*i*=1_^|*V*|^*P*′(*i*)log_2_*P*′(*i*), and *E*[MI(*U*, *V*)] represents the expectations of MI(*U*, *V*); MI(*U*, *V*) is expressed as follows:

(15) $$\begin{array}{c} \text{MI}\left(U,V\right)=\sum_{i=1}^{\left|U\right|}\sum_{j=1}^{\left|V\right|}P\left(i,j\right)\log_{2}\frac{P\left(i,j\right)}{P\left(i\right)P^{\prime }\left(j\right)}, \end{array}$$

where *P*(*i*)=|*U*_*i*_|/*n*, *P*′(*j*)=|*V*_*j*_|/*n*, *P*(*i*, *j*)=|*U*_*i*_∩*V*_*j*_|/*n*, *U*={*U*_*i*_|*i*=1,2,…, |*U*|}, and *V*={*V*_*j*_|*j*=1,2,…, |*V*|}. *U* and *V* represent two allocation methods for a dataset containing *n* elements, and *U*_*i*_ and *V*_*j*_ are clusters. In experimental verification, let *U* and *V* be the original labels and the clustering results of an algorithm, respectively. The value ranges of the two evaluation criteria are [−1,  1], and “1” denotes the best experimental result.

#### 3.1.2. Parameters of Each Algorithm

DF, the parameter of our proposal, was set from 0.01 to 0.05, in which 0.005 is the interval. And by an equal interval, we choose *n* dc from all *d*_*ij*_ in ascending order, where *n* is the number of samples of a given dataset. When performing DBSACN and OPTICS experiments, we took “(min(*d*_*ij*_) − max(*d*_*ij*_))/100” as the step and min(*d*_*ij*_) as the initial value to attain 100 epsilons, let the minpts be from 1 to 50, and choose the best result among five thousand clustering results. During the AP experiment, we set the initial value of the unique parameter “performance” of the AP algorithm to 1.5 times the maximum value of the similarity matrix, and each cycle is reduced by 0.03%; the optimal result is selected. The specific situation is shown in Table 2, where the DPC algorithm parameter is a suitable dc, and the PPC algorithm parameter is dc_percent. The results and arguments of DPC and PPC are obtained from [44].

#### 3.1.3. Code Sources and Preprocessing

To ensure that the experimental comparison is valid, we processed each dataset according to the method described in [25] and normalized the low-dimensional dataset and the DIM512 dataset. For preparing the Olivetti faces dataset, we first scaled each image (originally 92 × 112) to a smaller size of 15 × 15 and then performed principal component analysis (PCA) to filter out attributes of cumulative contribution rates greater than 90%. The normalization formula is as follows:

(16) $$\begin{array}{c} x_{ij}^{\prime }=\frac{x_{ij}-\min\left(x_{j}\right)}{\max\left(x_{j}\right)-\min\left(x_{j}\right)}, \end{array}$$

where *x*_*ij*_ represents the *j*^th^ value of the *i*^th^ data in the dataset *X* and max(*x*_*j*_) and min(*x*_*j*_) represent the maximum and minimum values of the  *j*^th^ feature in the dataset *X*, respectively.

The DBSCAN codes are all built-in functions of Matlab 2019a. The OPTICS code is from the pyclustering library, the AP code is from the sklearn library, and we provide the DPC-ZPS codes. We executed all methods on a personal computer with Windows 10, Intel(R) Core (TM) i7-8750H, 16 GB memory, and Matlab 2019a or Python 3.0.

### 3.2. Experimental Results and Analyses

**Figure 5:**
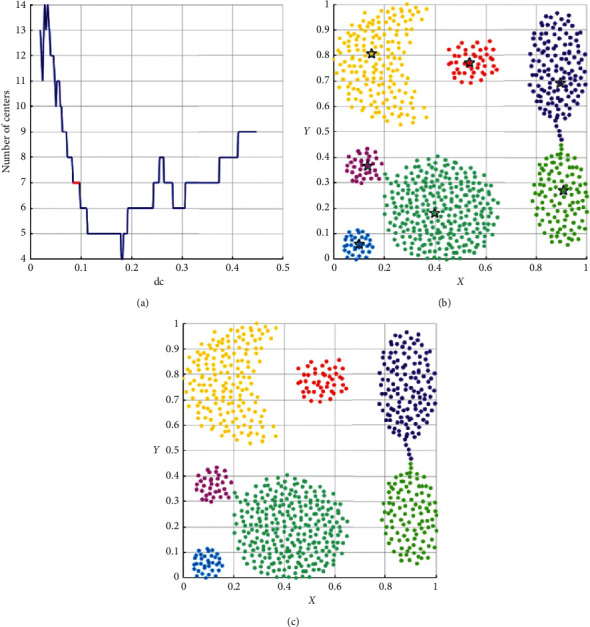
The analysis result of DPC-ZPS on the aggregation dataset: (a) relationship between dc and the number of centers; (b) DPC-ZPS on aggregation; (c) aggregation-ground truth.

**Figure 6:**
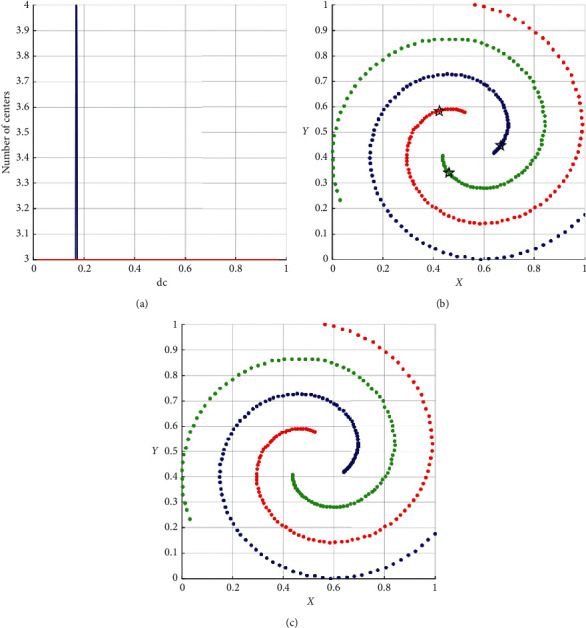
The analysis result of DPC-ZPS on the spiral dataset: (a) relationship between dc and the number of centers; (b) DPC-ZPS on spiral; (c) spiral-ground truth.

**Figure 7:**
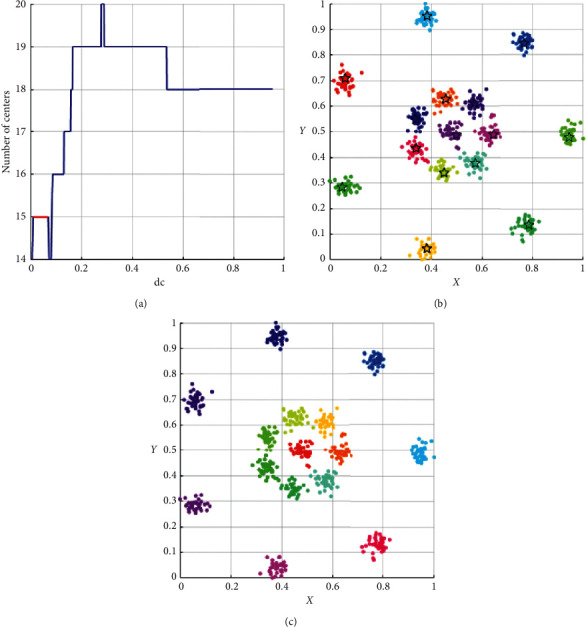
The analysis result of DPC-ZPS on the *D*31 dataset: (a) relationship between dc and the number of centers; (b) DPC-ZPS on *R*15; (c) *R*15 ground truth.

**Figure 8:**
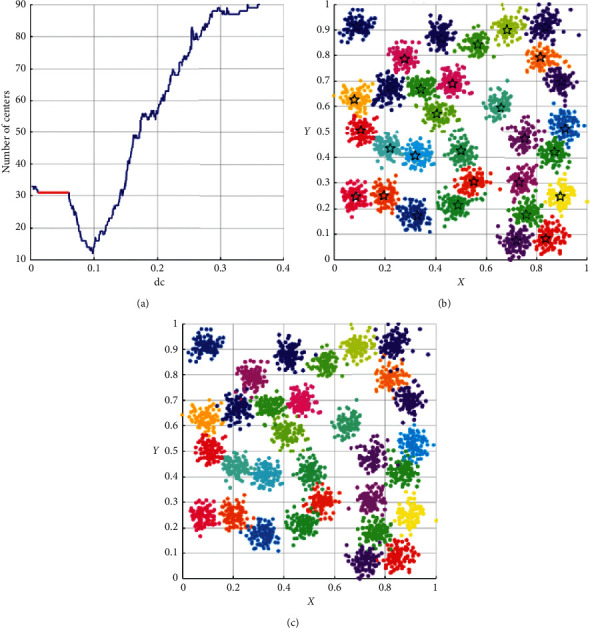
The analysis result of DPC-ZPS on the *D*31 dataset: (a) relationship between dc and the number of centers; (b) DPC-ZPS on *D*31; (c) *D*31 ground truth.

**Figure 9:**
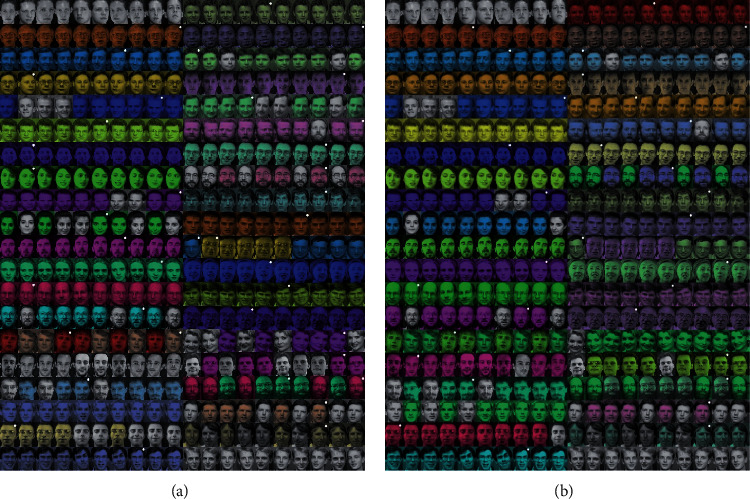
The clustering results on Olivetti faces by (a) DPC-ZPS and (b) DPC.

As shown in Table 3, the performance of DPC-ZPS is better than other control groups. Next, we will analyze the specific iterative process of our proposal from Figures 5–9. And each of the Figures 5–8 consist of three subgraphs. The left subgraphs represent the cutoff distance and the number of cluster centers determined by the DPC-ZPS algorithm, and the red line marks the suitable range of dc. The middle subgraph represents the clustering results of DPC-ZPS, and the right subgraph represents the category labels.  Figure 9 shows the clustering results of our method and the original DPC on the Olivetti face dataset.

As shown in Figure 5, our algorithm selects seven appropriate centers and successfully converges dc to the appropriate value interval through iteration. In the iterative processes, the change of center-num in the *L*-range is “14-8-7-7.” The number of centers remains unchanged, which means the seven clusters are relatively dependent. The final center-num of the *R*-range is “4,” so the clustering result of the *L*-range is selected as the final result.

**Figure 10:**
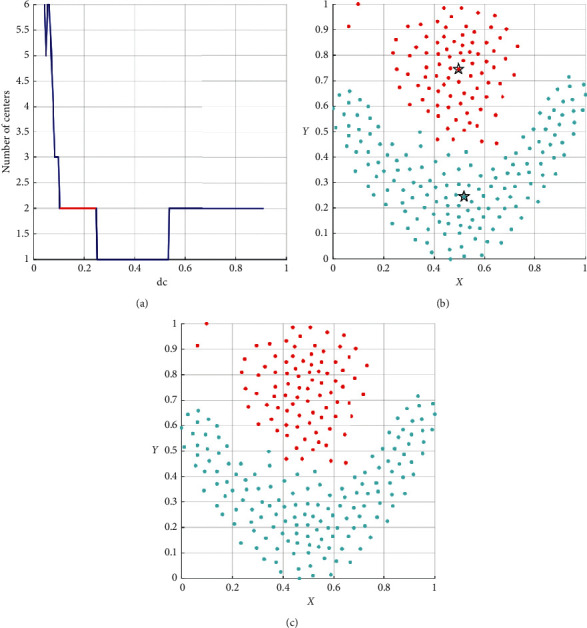
The analysis result of DPC-ZPS on the flame dataset: (a) relationship between dc and the number of centers; (b) DPC-ZPS on flame; (c) flame-ground truth.

In Figure 10(a), there is a min-range, and center-num is one. And in the *L*-range, the process of iteration is “6-2-2,” and that of the *R*-range is “2-1-1.” Therefore, the final clustering result lies in the *L*-range.

In the spiral dataset, three spiral clusters are far from each other. So in Figure 6(a), in most of the dc range, there are three suitable cluster centers. There is no *R*-range. And our method successfully merges all subclusters to three correct groups, which is consonant with Figure 6(c).

In the *L*-range of *R*15, the biggest center-num is 15, and the merge does not happen, while the last center-num of the *R*-range is 14. Hence, the actual clustering result is determined and is shown in Figure 7(b). The change process of *D*31 *L*-range is from 33 to 31. The ultima center number of the *R*-range is approximate to the minimum in Figure 8(a). Hence, the final cluster number is thirty-one.

The Olivetti faces dataset contains 40 (person) × 10 (photo) photos and is widely used in machine learning to test various algorithms. As shown in Table 3, the evaluation results of the DPC-ZPS on ARI are better than other algorithms.  Figure 9 shows the clustering results of the DPC-ZPS and DPC. The image marked with a white dot in the upper right corner is the cluster center, and the gray photos indicate that there are less than three elements in the cluster.

In Figure 9(b), there are no centers in the 4^th^, 6^th^, 8^th^, 10^th^, 11^st^ 18^th^, and 35^th^ group photos, which suggest that the traditional DPC algorithm may also incorrectly merge multiple clusters into one cluster. However, as shown in Figure 9, there are only the 16^th^ and 18^th^ group photos without centers. It demonstrates that DPC-ZPS is less likely to merge clusters incorrectly.

### 3.3. Discussion

Xie et al. [39, 40, 44] manifest that the selection rule of dc provided in [38] cannot meet various datasets. Table 2 shows that the values of dc and dc_percentage are diverse in diverse datasets, which increases the tuning cost and magnitude of difficulty, while in the six of the seven tested datasets, our argument is equal to 0.02.

**Figure 11:**
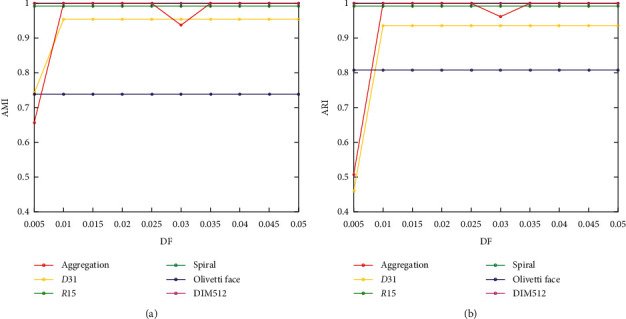
Results on different datasets with different depth factors.

The depth factor, the only parameter of the DPC-ZPS algorithm, is used in equation (6) to control the depth of the border between two adjacent clusters. When DF = 1, the neighboring cluster borders will contain all the elements in the two clusters. However, the edge should be composed of the elements with a shallow depth, so there are minimal parameter values in different datasets. Therefore, [0.005, 0.05] is a reasonable range for all of the tested datasets. As shown in Figure 11, most datasets severely fluctuate before DF = 0.015, which is just a small part of the whole; after that, our algorithm is not sensitive to the parameter changes. In addition, compared with the DPC and PPC algorithms, the DPC-ZPS algorithm does not require human intervention in the entire clustering process, which can overcome many defects caused by manual operation.

## 4. Conclusions

In this paper, to overcome the defects of human operation and the difficulty in determination of the suitable dc, we proposed the density peaks clustering by zero-pointed samples (DPC-ZPSs) of regional group borders. DPC-ZPS is based on the in-depth analyses of not only the changing rule between the dc and centers but also the relationship between the density of NCB and PCB. Our proposal covers two main parts: the merger strategy of subclusters based on the cluster borders and the iteration strategy. The merger strategy adaptively determines the threshold of merge for each pairwise local cluster. And the iterative process is to find a suitable range of dc automatically. And experimental results indicate our method is more accurate without artificial operation and has a more reasonable and less sensitive threshold value range. Additionally, we will use the natural nearest neighbors to optimize the local density measurement and assignment process.

## Figures and Tables

**Table 1:** Detailed information on tested datasets.

Dataset	No. of records	No. of attributes	No. of clusters	Source
Aggregation	788	2	7	[48]
Flame	240	2	2	[49]
Spiral	312	2	3	[50]
D31	3100	2	31	[51]
R15	600	2	15	[51]
DIM512	1024	512	16	[52]
Olivetti faces	400	92 × 112	40	[53]

**Table 2:** Parameters setting.

Dataset	DPC-ZPS	PPC	DPC	DBSCAN	OPTICS	AP
Aggregation	0.02	0.012	0.034	0.0643/14	0.06/10	−0.96
Flame	0.03	0.027	0.028	0.1177/14	0.10/8	−2.19
Spiral	0.02	0.01	0.018	0.0418/1	0.04/1	−1.73
R15	0.02	0.015	0.006	0.0508/30	0.004/11	−0.17
D31	0.02	0.006	0.006	0.0377/37	0.03/23	−0.08
DIM512	0.02	0.039	0.006	0.36/2	0.19/1	−1
Olivetti face	0.02	0.001	0.004	0.0294/2	0.59/2	−0.247

**Table 3:** Clustering results.

Dataset	Evaluation criteria	DPC-ZPS	PPC	DPC	DBSCAN	OPTICS	AP
Aggregation	AMI	**1.0000**	0.9922	**1.0000**	0.9785	0.9368	0.7352
	ARI	**1.0000**	0.9956	**1.0000**	0.9888	0.9747	0.6427

Flame	AMI	**1.0000**	**1.0000**	**1.0000**	0.8844	0.7385	0.3239
	ARI	**1.0000**	**1.0000**	**1.0000**	0.9550	0.8965	0.3950

Spiral	AMI	**1.0000**	**1.0000**	**1.0000**	**1.0000**	**1.0000**	−0.0014
	ARI	**1.0000**	**1.0000**	**1.0000**	**1.0000**	**1.0000**	−0.0016

*D*31	AMI	**0.9556**	0.9554	0.9554	0.9087	0.7901	0.8563
	ARI	**0.9367**	0.9365	0.9365	0.8450	0.5814	0.7991

*R*15	AMI	**0.9938**	**0.9938**	**0.9938**	0.9916	0.9734	0.9907
	ARI	**0.9928**	**0.9928**	**0.9928**	0.9893	0.9785	0.9891

DIM512	AMI	**1.0000**	**1.0000**	**1.0000**	**1.0000**	0.9029	**1.0000**
	ARI	**1.0000**	**1.0000**	**1.0000**	**1.0000**	0.9432	**1.0000**

Olivetti face	AMI	0.8086	**0.8447**	0.8259	0.7106	0.4286	0.7297
	ARI	**0.7385**	0.7155	0.6863	0.4668	0.5036	0.6260

## Data Availability

All datasets in this paper are from UCI. All readers are able to access datasets from it.
